# The complete chloroplast genome sequence of *Rubus peltatus* Maxim. (Rosaceae)

**DOI:** 10.1080/23802359.2022.2085529

**Published:** 2022-06-29

**Authors:** Fei Qiao, Wei Guo, Wenkui Dai, Weicheng Huang, Ling Wu

**Affiliations:** a Department of Horticulture and Landscape Architecture, Zhongkai University of Agriculture and Engineering, Guangzhou, China; b Guangdong Forestry Administrative Affairs Service Center, Guangzhou, China

**Keywords:** *Rubus peltatus* Maxim., Rosaceae, complete chloroplast genome, automated assembly

## Abstract

*Rubus peltatus* Maxim. (Bull. Acad. Imp. 1871) is a wild species endemic to East and Southeast China. However, genetic resources were unavailable for this species. It holds great potential for domestication or other breeding purposes with the extraordinary large yellow fruits. The complete chloroplast genome sequence of *R. peltatus*, assembled with Illumina Hiseq X Ten platform sequencing data, was reported. The chloroplast genome was 155,582 bp in length. The large single-copy (LSC) and small single-copy (SSC) of 85,329 bp and 18,779 bp were separated by two inverted repeats (IRs) of 25,737 bp. The chloroplast genome of *R*. *peltatus* contains 130 genes, including eight transfer RNA genes, 36 ribosomal RNA genes, and 86 protein-coding genes. Phylogenetic analysis supports *R*. *peltatus* has a close relationship with the *R. cochinchinensis* and *R. takesimensis.*

*Rubus* Linnaeus is a conspicuous genus in the family of diverse raspberries and blackberries, which are popular fruits around the world with the low calories and excellent sources of fiber, vitamin C, and antioxidant compounds. Being the diversity center for the *Rubus* genus, China has about 204 species, accounting for 97% of Asian species (Lu 1983). However, few wild *Rubus* species has been domesticated or utilized so far. The species *Rubus peltatus* Maxim. is endemic to East and Southeast China, and is promising for domestication with extraordinary large yellow fruits. However, genetic resources were unavailable for this species. In this study, we reported and characterized the chloroplast genomes of *R. peltatus* for further study and utilization of this species.

Plant samples of *R. peltatus* were collected in the field from Guizhou province, China (27°54′N, 108°34′E, 1018 m above sea level). The voucher specimen was deposited at the Herbarium of Sun Yat-sen University (SYS) with accession number SYS00001512 (Weicheng Huang, huangwc0921@163.com). Total genomic DNA was extracted from silica gel dried leaves using a modified CTAB protocol (Doyle 1991). After agarose gel electrophoresis for inspection of integrity and quantification, the total DNA was sent to Berry Genomics Bio-Technique Co. Ltd. (Beijing, China) for sequencing service. A fragment library with insertion size of 500 bp was prepared and sequenced using paired-end reads 150 bp in length on the Illumina Hiseq X Ten platform (Illumina, Inc., San Diego, CA). Using fastp v0.20.1 (Chen et al. 2018), raw-reads were subject to adapter cutting without further trimming or filtering as recommended by the *de novo* assembler NOVOPlasty (Dierckxsens et al. 2017). Next, setting the whole chloroplast genome sequences of *Rubus leucanthus* (MK105853.1) as seed, we assembled the *R. peltatus* chloroplast genome using an extension algorithm implemented in NOVOPlasty with default settings. The assembled cp genome was annotated using GeSeq (Tillich et al. 2017), and the annotation was corrected using Geneious v.9.0.2 (Kearse et al. 2012). The complete genome sequence and annotations of *R. peltatus* were submitted to GenBank under accession number NC_056937.1 (Wei Guo, gwei717@163.com).

The complete cp genome of *R. peltatus* was 155,582 bp in length. The typical structure consists of a large single-copy (LSC) of 85,329 bp and a small single-copy (SSC) of 18,779 bp, separated by two inverted repeats (IRs) (25,737 bp). The chloroplast genome circle was joined end-to-end in the order ‘LSC-IRA-SSC-IRB’. The overall GC content of cp genome was 36.91%. There were 130 predicted genes, 86 protein-encoding genes, 36 tRNA genes, and eight rRNA genes.

To confirm the phylogeny position of *R. peltatus* in Rosaceae, 19 representative species within family Rosaceae were obtained from GenBank database (https://www.ncbi.nlm.nih.gov/). *Cucumis sativus* (DQ119058.1) was specified as one outgroup taxon. The sequences were aligned using MAFFT v.7.313 (Katoh and Standley 2013). A maximum-likelihood tree was constructed in the package RAxML v.8.2.11 (Stamatakis 2014) using *Cucumis sativus* as outgroup with bootstrap replicates of 100 (Figure 1). Phylogenetic analysis demonstrated that *R. peltatus* was placed in the Rubeae tribe with a closer relationship with *R. cochinchinensis* and *R. takesimensis*, than that with *R. leucanthus* or *R. rufus*.

**Figure 1.**
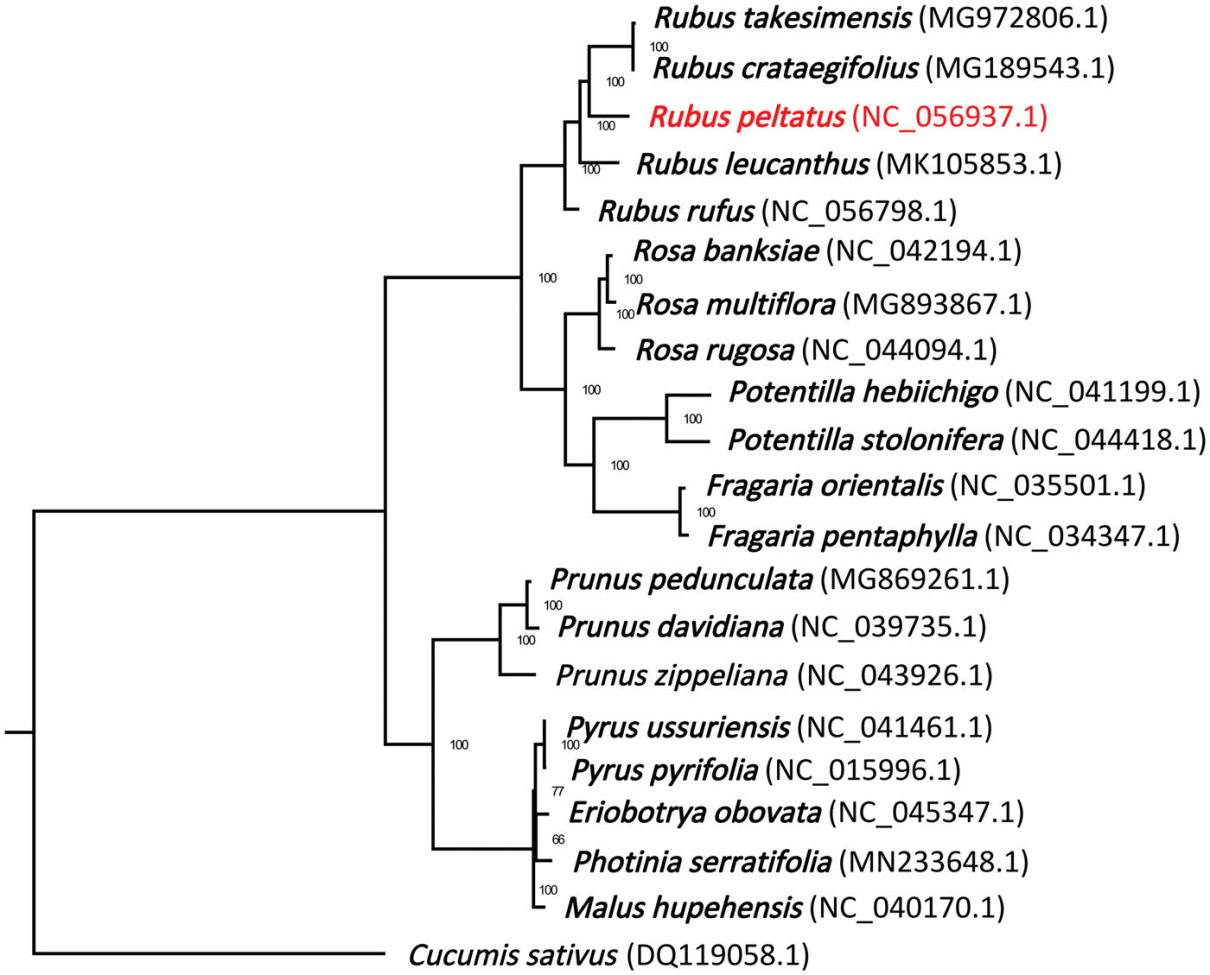
Maximum-likelihood tree of Rosaceae based on complete chloroplast genome, with *Cucumis sativus* as outgroup. The *Rubus peltatus* Maxim. is marked in red and bootstrap support values are shown next to the nodes.

## Data Availability

The genome sequence data that support the findings of this study are openly available in GenBank of NCBI at https://www.ncbi.nlm.nih.gov/ under the accession no. NC_056937.1. The associated BioProject, SRA, and Bio-Sample numbers are PRJNA747400, SRR12424495, and SAMN15763317, respectively.
